# TR-FRET-Based Immunoassay to Measure Ataxin-2 as a Target Engagement Marker in Spinocerebellar Ataxia Type 2

**DOI:** 10.1007/s12035-023-03294-y

**Published:** 2023-03-09

**Authors:** Jessica Bux, Nesli Ece Sen, Isa-Maria Klink, Stefan Hauser, Matthis Synofzik, Ludger Schöls, Georg Auburger, Olaf Riess, Jeannette Hübener-Schmid

**Affiliations:** 1 Institute of Medical Genetics and Applied Genomics, University of Tübingen, Tübingen, Germany; 2 Centre for Rare Diseases, Medical Faculty, University of Tübingen, Tübingen, Germany; 3 Experimental Neurology, Goethe University, Frankfurt am Main, Germany; 4 Department of Molecular and Cellular Biology, Faculty of Sciences III, University of Geneva, Geneva, Switzerland; 5 Department for Neurodegenerative Diseases and Hertie-Institute for Clinical Brain Research, University of Tübingen, Tübingen, Germany; 6 German Center for Neurodegenerative Diseases (DZNE), Tübingen, Germany; 7 NGS Competence Center Tübingen, Tübingen, Germany

**Keywords:** Spinocerebellar ataxia type 2, Ataxin-2, Biomarker, Time-resolved fluorescence energy transfer, Target engagement

## Abstract

Spinocerebellar ataxia type 2 (SCA2) is an autosomal dominantly inherited neurodegenerative disease, which belongs to the trinucleotide repeat disease group with a CAG repeat expansion in exon 1 of the *ATXN2* gene resulting in an ataxin-2 protein with an expanded polyglutamine (polyQ)-stretch. The disease is late manifesting leading to early death. Today, therapeutic interventions to cure the disease or even to decelerate disease progression are not available yet. Furthermore, primary readout parameter for disease progression and therapeutic intervention studies are limited. Thus, there is an urgent need for quantifiable molecular biomarkers such as ataxin-2 becoming even more important due to numerous potential protein-lowering therapeutic intervention strategies. The aim of this study was to establish a sensitive technique to measure the amount of soluble polyQ-expanded ataxin-2 in human biofluids to evaluate ataxin-2 protein levels as prognostic and/or therapeutic biomarker in SCA2. Time-resolved fluorescence energy transfer (TR-FRET) was used to establish a polyQ-expanded ataxin-2-specific immunoassay. Two different ataxin-2 antibodies and two different polyQ-binding antibodies were validated in three different concentrations and tested in cellular and animal tissue as well as in human cell lines, comparing different buffer conditions to evaluate the best assay conditions. We established a TR-FRET-based immunoassay for soluble polyQ-expanded ataxin-2 and validated measurements in human cell lines including iPSC-derived cortical neurons. Additionally, our immunoassay was sensitive enough to monitor small ataxin-2 expression changes by siRNA or starvation treatment. We successfully established the first sensitive ataxin-2 immunoassay to measure specifically soluble polyQ-expanded ataxin-2 in human biomaterials.

**Supplementary Information:**

The online version contains supplementary material available at 10.1007/s12035-023-03294-y.

## Introduction

Spinocerebellar ataxia type 2 (SCA2) is an autosomal dominantly inherited neurodegenerative disease which is caused by the expansion of the triplet CAG in exon 1 of the *ATXN2* gene localized on chromosome 12q24 [1, 2]. The triplet CAG encodes for the amino acid glutamine (Q) and therefore, SCA2 belongs to the group of polyglutamine (polyQ) expansion diseases like Huntington’s disease (HD); SCA1, 3, 6, 7, 17; dentatorubral-pallidoluysian atrophy (DRPLA); and spinal and bulbar muscular atrophy (SBMA). Normal repeat length varies between 14 and 29 glutamines (most individuals have 22Q) [3], and intermediate length alleles (27–33Q) are associated with amyotrophic lateral sclerosis (ALS) [4] and a length of more than 32Q results in SCA2 [5], while most SCA2 patients show expanded repeats between 36 and 52, respectively [6, 7], and rare expansions beyond 800Q were observed [8].

For SCA2, mean age of onset is about 32 years. Age of onset, disease duration but also clinical manifestation show substantial variability. Key features include cerebellar ataxia and slowing of saccadic eye movements in variable combination with cerebellar dysarthria, dysphagia, peripheral neuropathy, and postural tremor. Degeneration of Purkinje cells in the cerebellum is the main neuropathological hallmark of SCA2 [7, 9–21].

The protein ataxin-2 (ATXN2) is predominantly localized in the cytosol [22] and is leading with disease progression to intranuclear inclusions in the brainstem [23] and to cytoplasmic “micro”aggregates in cerebellar Purkinje cells [24]. ATXN2 has a role in posttranscriptional RNA modification, quality control, and translation [25–29]. Under stress conditions, the expression and translation of ATXN2 is enhanced, and the protein relocalizes into stress granules [30].

So far, there is no curative therapy for SCA2 [31]. Clinical studies are difficult as primary readout parameters reflecting disease activity or key pathogenic processes are limited. Clinical scores like the Scale for the Assessment and Rating of Ataxia (SARA) are commonly used to monitor disease progression but show a high inter-rater and day-to-day variability [32]. Neurofilament light (NfL) is the best-established fluid biomarker for measuring neurodegeneration in several neurodegenerative diseases but with poor specificity [33]. For SCA2, NfL levels were associated with disease severity and cerebellar atrophy as well as get increased already before disease onset in pre-ataxic mutation carriers [34–36]. Another biofluid marker which shows potential as neurodegenerative marker in different neurological disorders including SCA2 is tau, a protein which promotes microtubule assembly and stability and is released under neuroaxonal damage. In SCA2, tau was significantly elevated in CSF compared to controls [37]. Additionally, imaging analyses showed olivopontocerebellar atrophy and pontine brainstem volume loss before disease onset and thalamus as well as parietal cortical atrophy at higher disease stages [38–40]. With the development of disease protein-lowering therapies like siRNA or antisense oligonucleotides (ASO) in polyQ diseases, the soluble disease protein itself represents a promising target-based molecular marker to measure protein lowering under therapies (target engagement). Therefore, for several polyQ diseases like HD [41–43] or SCA3 [44, 45], TR-FRET-based immunoassays for quantification of total and polyQ-expanded disease proteins, like huntingtin or ataxin-3, have been established. Additionally, to detect very low protein concentrations in peripheral blood or CSF, ultrasensitive single-molecule counting (SMC) immunoassays were validated [46, 47] and used as prognostic and/or therapeutic readout parameters. In SCA2, the disease protein ATXN2 could also represent a potential prognostic and/or therapeutic biomarker, as the amount of polyQ-expanded ATXN2 in, e.g., blood or CSF, may reflect the course of the disease and potential therapeutic success.

Intermediated repeat expansions in ATXN2 (27-32Qs) are viewed as a genetic risk factor, as they modulate the pathogenesis and the age at onset of several other neurodegenerative diseases including transthyretin familial amyloid polyneuropathy (TTR-FAP), frontotemporal dementia (FTD), Alzheimer´s disease (AD), corticobasal syndrome, ALS, and SCA3 [48–54]. Furthermore, CAA interruptions within the CAG repeat are claimed to have an influence on the pathogenesis and the phenotype of neurological diseases [48]. It is assumed that CAA interruptions are stabilizing the CAG repeat and therefore may explain why intermediated repeats are associated with, e.g., ALS and other neurological disorders but did not result in a mild type of SCA2 [55]. Whereas SCA2 patients demonstrate only an uninterrupted CAG repeat within ATXN2, a patient with corticobasal syndrome was reported which demonstrated 27/39 CAG repeats within ATXN2 with a CAG repeat interrupted by three (intermediated allele) or four (expanded allele) CAA motifs. This patient did not suffer from SCA2 [56]. Recently, a 9-bp duplication within ATXN2 was identified which led to significant decrease in the age at onset in both SCA3 and C9orf72-ALS [57].

Therefore, the establishment of sensitive immunoassays to determine total and polyQ-expanded ATXN2 in human biofluids is not only of interest for the rare disease SCA2 but also for other (rare) neurological disorders. In the current study, it was our aim to provide a first step for the development of sensitive immunoassays for the quantitative determination of soluble polyQ-expanded ATXN2 protein in patient-derived cell lines and mammalian biomaterials.

## Materials and Methods

### Ethical Use of Animals

All mice were maintained by animal care staff and veterinarians of the University of Frankfurt/Main Zentrale Forschungs-Einrichtung (ZFE). All procedures were performed according to the German Animal Welfare Act and the guidelines of the Federation of European Laboratory Animal Science Associations, based on European Union legislation (Directive 2010/63/EU). Animal experiments were approved by the local ethics committee (Regierungs-Präsidium Darmstadt V54-19c18-FK/1083).

### Ethical Use of Human Tissue

All the work involving human tissue has been carried out in accordance with the Code of Ethics of the World Medical Association (Declaration of Helsinki) and with national legislation as well as our institutional guidelines. Experiments were approved by the local ethics committee (ethical vote Tübingen, 598/2011BO1 and 911/2019BO2).

### Mouse and iPSC Sample Description

Different mouse tissues (liver, hemisphere, cerebellum) isolated from young (2.5–3 months) and old (14–18 months) knock-in mouse lines modeling SCA2, including *ATXN2-*CAG42 [58] and *Atxn2-*CAG100 knock-in mice [59], were used during the establishment of the assay conditions. The generation of mouse embryonic fibroblast (MEF) lines from *Atxn2-*CAG100 knock-in mice was reported before [59]. Human fibroblasts were isolated from skin biopsies of three SCA2 patients and three neurologically healthy controls after written informed consent. Fibroblasts were reprogrammed into induced pluripotent stem cells (iPSCs) as specified earlier [60] and differentiated into cortical neurons (CNs) as described before [61]. Specification of analyzed human cell lines are represented in Table 1. Human serum samples from four SCA2 patients and five neurologically healthy controls were isolated using serum preparation tubes (BD Biosciences, Heidelberg, Germany).

**Table 1:** Specification of human cell lines used in this study

Line	Number of iPSC clones	*ATXN2* CAG repeat length	Age at biopsy	Sex
Co-1	1	21/21	74 years	Male
Co-2	1	14/21	69 years	Female
Co-3	1	14/23	46 years	Female
Co-4	1	21/23	46 years	Male
Ax-1	2	23/36	31 years	Male
Ax-2	1	22/42	36 years	Female
Ax-3	1	22/38	45 years	Female

### Cell Culture and Transfection

MEF and human embryonic kidney (HEK293T) cells were maintained in DMEM (Thermo Fisher Scientific, Waltham, USA) supplemented with 10% fetal bovine serum (FBS) and 1% antibiotics-antimycotics (both Thermo Fisher Scientific, Waltham, USA) at 5% CO_2_ and 37 °C. For MEF, experiments were carried out only within the first 5 passages. Different ATXN2 plasmids including GFP-ATXN2-22Q/myc-ATXN2-22Q, GFP-ATXN2-79Q/myc-ATXN2-79Q, or GFP-empty/myc-empty as internal control were used for transfection experiments.

HEK293T cells were transfected with GFP or myc ATXN2 plasmids with different polyQ length (GFP-22Q/myc-22Q, GFP-79Q/myc-79Q, or GFP-empty/myc-empty as internal control) with Attractene transfection reagent (Qiagen, Hilden, Germany) using the traditional transfection protocol. Shortly, 24 h before transfection, 400,000 HEK293T cells per well were seeded on a six-well tissue culture plate in DMEM medium. For transfection, 1.2 μg plasmid DNA, 100 μl OptiMEM (Thermo Fisher Scientific, Waltham, USA), and 4.5 μl Attractene Transfection Reagent were mixed, incubated for 15 min at room temperature (RT), and afterwards dropped carefully on the seeded cells. Seventy-two hours after transfection, HEK293T cells as well as MEF cells were harvested with DPBS (Thermo Fisher Scientific, Waltham, USA), centrifuged for 5 min at 300*g*, and directly lysed in RIPA buffer (50 mM Tris, pH 8.0, 150 mM NaCl, 0.1% SDS, 0.5% sodium deoxycholate, 1% IGEPAL) supplemented with cOmplete (EDTA-free) Protease Inhibitor (Roche, Mannheim, Germany) followed by incubation on ice for 30 min and vortexing every 10 min. For lysate preparation, the cell homogenates were centrifuged for 30 min at 4 °C and 16,100*g*. The Bradford protein assay was used to determine the total protein concentration [62].

### Cell Culture Starvation and siRNA Experiments

For the starvation experiments, 400,000 HEK293T cells per well were seeded on a six-well tissue culture plate and transfected with ATXN2 GFP-22Q, GFP-79Q or GFP-empty as control, as described above.

Seventy-two hours after transfection, the medium was changed to Hanks’ Balanced Salt Solution (Thermo Fisher Scientific, Waltham, USA) and incubated for either 0 h, 1 h, or 2 h, respectively. After incubation, cells were harvested as described.

For esiRNA experiments, knockdown of ATXN2 was achieved using endoribonuclease-prepared siRNA (esiRNA) directed against human ATXN2 (MISSION® esiRNA EHU104101, Sigma-Aldrich, Missouri, USA) or control esiRNA against *Renilla* luciferase (MISSION® esiRNA EHURLUC, Sigma-Aldrich, Missouri, USA). Four hundred thousand HEK293T cells per well were seeded on a six-well tissue culture plate. The transfection followed the traditional Attractene transfection protocol (Qiagen, Hilden, Germany) with 1.2 μg plasmid DNA (ATXN2 GFP-22Q, ATXN2 GFP-79Q or GFP-empty as control), 18 pmol esiRNA, and 4.5 μl Attractene Transfection Reagent. Seventy-two hours after transfection, cells were harvested with DPBS, centrifuged for 5 min at 300*g* and lysed in RIPA for 30 min on ice and vortexing every 10 min.

### Isolation of Recombinant ATXN2

HEK293T cells (1.2 × 10^7^) were seeded per 175 cm^2^ cell culture dish. After 24 h, cells were transfected with 8 μg ATXN2 Myc-22Q or Myc-79Q constructs using polyethylenimine (PEI) transfection reagent (Polysciences, Warrintong, USA). Seventy-two hours after transfection, cells were harvested in M-Per Mammalian Protein Extraction Reagent (Thermo Fisher Scientific, Waltham, USA) followed by lysis of adherent mammalian cells and procedure for IP of Myc-tagged proteins using Pierce Anti-Myc Agarose as described in the instruction manual (Thermo Fisher Scientific, Waltham, USA). Recombinant proteins were eluted using Pierce IgG Elution buffer (Thermo Fisher Scientific, Waltham, USA).

### Protein Extraction

For the establishment of the TR-FRET, mouse tissues and cells were homogenized in three different lysis buffers: RIPA buffer (50 mM Tris, pH 8.0, 150 mM NaCl, 0.1% SDS, 0.5% sodium deoxycholate, 1% IGEPAL), TES buffer (20 mM Tris, pH 7.5, 2 mM EDTA, 100 mM NaCl) supplemented with TNES buffer (50 mM Tris pH 7,5, 2 mM EDTA, 100 mM NaCl, 1% IGEPAL), or PBS buffer (DPBS (1×) Dulbecco’s Phosphate-Buffered Saline with 1% Triton™ X-100). All buffers were supplemented with cOmplete (EDTA-free) Protease Inhibitor (Roche, Mannheim, Germany). For each buffer condition, 10 mg tissue or cells were homogenized mechanistically in 100 μl of the respective buffer using the VDI 12 homogenisator (VWR, Darmstadt, Germany). Afterwards, homogenates were incubated on ice for 30 min vortexing every 10 min. Homogenates were stored at − 80 °C for later TR-FRET analyzes. Part of the homogenates were centrifuged at 13 200*g* for 30 min. Lysates were transferred to new collection tubes supplemented with 10% glycerol and stored at − 80 °C for later western blot analyses. The Bradford protein assay was used to determine the total protein concentration from homogenates and lysates [62].

### SDS-PAGE and Western Blot

Twenty to 30 μg of total protein from cell culture or mouse tissue lysates were supplemented with 4 × LDS sample buffer (1 M Tris pH 8.5, 2 mm EDTA, 8% LDS, 40% glycerol, 0.075% CBB G, 0.025% phenol red) in a ratio 3:1 and 0.1 M dithiothreitol. Samples were heat denatured for 10 min at 70 °C and afterwards, electrophoretically separated [63] using 8 to 10% Bis-Tris gels with the electrophoresis MOPS buffer (50 mM MOPS, 50 mM Tris pH 7.7, 0.1% SDS, 1 mM EDTA) at 100 V, 250 mA for 2–2.5 h. Proteins were blotted [64] on nitrocellulose membranes (Amersham Protran Premium 0.2 μm, GE Healthcare) using transfer buffer (25 mM Bicine, 25 mM Bis-Tris pH 7.2, 1 mM EDTA, 15% methanol) at 80 V and 250 mA for 1.5 h. After transfer, the membranes were blocked with 5% skim milk powder in Tris-buffered saline (TBS) for 1 h, washed with TBS-T (TBS with 0.1% Tween 20) and incubated overnight at 4 °C with the following primary antibodies diluted in TBS-T: ataxin-2 polyclonal antibody (1:1000, 21776-1-AP, rabbit, Proteintech Group, Rosemont, USA); mouse anti-β-actin monoclonal antibody (1:500, clone AC-15, Sigma-Aldrich, Darmstadt, Germany). Afterwards, membranes were washed with TBS-T and incubated at RT for 1 h with secondary IRDye antibodies goat anti-mouse 800CW or goat anti-rabbit 800CW (both 1:5000, Li-Cor Biotechnology GmbH, Bad Homburg, Germany), respectively. After washing with TBS-T, fluorescence signals were detected using the LI-COR ODYSSEY FC and quantified with Image Studio 4.0 software (both Li-Cor Biotechnology GmbH, Bad Homburg, Germany). All full western blot images are provided as supplementary information figure 3a-f.

### TR-FRET

To establish a polyQ-expanded ATXN2 specific time resolved fluorescence energy transfer (TR-FRET)-based immunoassay, two different ATXN2 antibodies and two different polyQ-specific antibodies were compared. Therefore, the two ATXN2 specific antibodies ataxin-2 polyclonal antibody (21776-1-AP, Proteintech Group, Rosemont, USA) and purified mouse anti-ataxin-2 monoclonal antibody (AB_398900, Becton, Dickinson and Company, Sparks, USA) were labeled with the fluorophore Tb by the company CisBio Inc. (PerkinElmer, Waltham, USA). Additionally, the two polyQ-specific antibodies clone MW1 (AB 528290, Development Studies Hybridoma Bank, Iowa, USA), and clone 5TF1-1C2 (MAB1574, Sigma-Aldrich, Darmstadt, Germany) received the acceptor fluorophore D2 by the same company. For establishment of the TR-FRET-based immunoassay, the labeled antibodies were diluted in detection buffer (50 mM NaH_2_PO_4_, 400 mM NaF, 0.1% BSA, 0.05% Tween-20) in different concentrations (Tb-labeled antibodies: 0.3 ng, 0.5 ng, 1 ng and D2-labeled antibodies: 1 ng, 3 ng, 10 ng). Mouse tissue or cell culture homogenates were diluted to a total protein concentration of either 2 μg/μl or 1 μg/μl in one of the following buffers (RIPA, TES/TNES or PBS) supplemented with cOmplete (EDTA-free) Protease Inhibitor. Human serum samples were measured undiluted or diluted 1:2 or 1:4 with PBS supplement with cOmplete (EDTA-free) Protease Inhibitor, respectively. Five microliters per diluted sample was incubated in duplicates with 1 μl antibody-mix (Tb-antibody with D2-antibody, ratio 1:1) in a low-volume white ProxiPlate 384 TC Plus plate (PerkinElmer, Waltham, USA) for 24 h at 4 °C. Signals were detected at 620 nm and 665 nm with the Multimode Plate Reader Envision (PerkinElmer, Waltham, USA), and the ratio between 665/620 was normalized to the total protein concentration and over the background signal (deltaF).

### Statistical Analysis

The program GraphPad Prism 8 (GraphPad Software Inc., San Diego, USA) was used for the statistical evaluation of all data and for data visualization. Limit of detection (LOD) and quantification (LOQ) were calculated using GraphPad Prism software with a nonlinear regression model using a two-site binding saturation curve fit (including specific and background signal). The LOD is defined as the lowest concentration giving a signal greater than the background signal (above 3 standard deviations). The LOQ is defined as the lowest concentration giving a signal greater than the background signal (+ 10 standard deviations).

Normality of the datasets was evaluated by Shapiro-Wilk test. Due to the non-normally distributed data, the non-parametric Mann-Whitney *U* test was used to compare the different groups. *P*-values with less than 0.05 were considered statistically significant with *p < 0.05, **p < 0.01, and ***p < 0.001. All values are shown as mean ± standard error of the mean, SEM.

## Results

### Establishment of Antibody Combination and Concentration

**Fig. 1:**
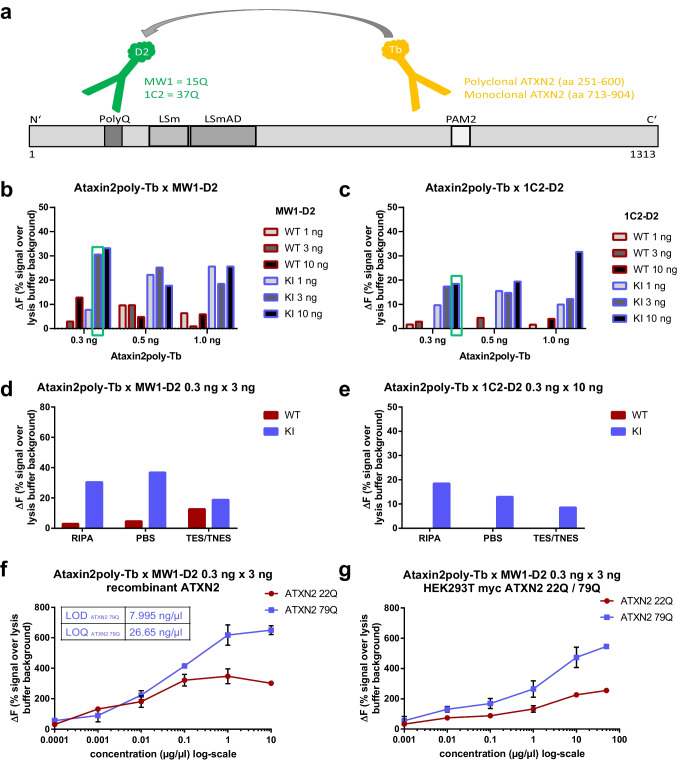
Establishment of a polyQ-expanded ATXN2 TR-FRET-based immunoassay. **a** Schematic illustration of the functional mechanism of a TR-FRET-based immunoassay to detect polyQ-expanded ATXN2. The ATXN2 protein with its four most important domains (PolyQ, LSm, LSmAD and PAM2) is shown to visualize the binding sites of the tested antibodies. Antibodies used as donor were labeled with the luminophore terbium cryptate (Tb) and the acceptor antibodies with the luminophore D2. Two D2-labeled antibodies (MW1-D2 and 1C2-D2) and two ATXN2-specific antibodies (ataxin2poly-Tb and Ataxin2mono-Tb) were tested. **b**, **c** Combination of the antibodies ataxin2poly-Tb and MW1-D2 (**b**) or ataxin2poly-Tb and 1C2-D2 (**c**) each in three different concentrations: ataxin2poly-Tb with 0.3 ng, 0.5 ng and 1 ng/μl, MW1- D2 or 1C2-D2 with 1 ng, 3 ng, and 10 ng/μl. Conduction of the TR-FRET measurement with homogenates from wildtype mouse liver (red; WT) and Atxn2-CAG100 knock-in mouse liver (blue; KI) lysed in RIPA buffer, using a total protein concentration of 1 μg/μl. The highest discrimination between WT and KI (green box) was achieved with the concentrations of ataxin2poly-Tb 0.3 ng/μl × MW1-D2 3 ng/μl (**b**) and ataxin2poly-Tb 0.3 ng/μl x 1C2-D2 10 ng/μl (**c**). **d**, **e** Comparison of three different lysis buffers including RIPA, PBS, and TES/TNES. TR-FRET measurement with homogenates from wildtype mouse liver (red; WT) and Atxn2-CAG100 knock-in mouse liver (blue; KI) in the established antibody concentrations: ataxin2poly-Tb 0.3 ng/μl × MW1-D2 3 ng/μl (**d**) and ataxin2poly-Tb 0.3 ng/μl × 1C2-D2 10 ng/μl (**e**). **f**, **g** Detection of purified recombinant ATXN2 proteins (**f**) or protein homogenates overexpressing ATXN2 of various lengths (**g**) with the antibody combination ataxin2poly-Tb 0.3 ng/μl × MW1-D2 3 ng/μl showed specificity for polyQ-expanded ATXN2 (ATXN2 79Q) over normal ATXN2 (ATXN2 22Q). The assay’s limit of detection (LOD) was defined as the concentration corresponding to the signal 3 SDs above the lysis buffer background (zero calibrator) and upper limit of quantification (LOQ) as the concentration corresponding to the signal 10 SDs above the lysis buffer background. LOD = 7.995 ng/μl and LOQ = 26.65 ng/μl for recombinant ATXN2 79Q

To establish a TR-FRET-based immunoassay to quantify specifically soluble polyQ-expanded ATXN2 protein, two different Tb-labeled ATXN2 specific antibodies (polyclonal ATXN2 antibody (ataxin2poly-Tb, aa 251-600) and monoclonal ATXN2 antibody (Ataxin2mono-Tb, aa 713-904)) as well as two different D2-labeled polyQ-specific antibodies (MW1-D2 (≥ 15Q) and 1C2-D2 (≥ 37Q)) were tested in combination with each other, each at three different concentrations (for Tb 0.3 ng, 0.5 ng and 1 ng/μl, for D2 1 ng, 3 ng, and 10 ng/μl) (schematic representation in Fig. 1a). If both antibodies bind specifically and in close proximity, an energy transfer takes place between the donor (Tb) and the acceptor (D2). The measured signal is proportional to the ATXN2 protein concentration of the tested sample and needs to be normalized to the total protein concentration and over the background signal. To evaluate the different antibody combinations, liver homogenates from *Atxn2*-CAG100 knock-in mice [59] (blue; KI) compared to wildtype mice (red; WT) and homogenates of MEF from *Atxn2*-CAG100 knock-in mice [59] compared to wildtype mice were generated in RIPA buffer. For the antibody combination ataxin2poly-Tb × MW1-D2, the best discrimination between WT and KI was achieved with the concentration of ataxin2poly-Tb 0.3 ng/μl × MW1-D2 3 ng/μl (labeled in green, Fig. 1b). Antibody combination ataxin2poly-Tb x 1C2-D2 showed the best discrimination between WT and KI with the concentration of ataxin2poly-Tb 0.3 ng/μl × 1C2-D2 10 ng/μl (labeled in green, Fig. 1c). Evaluation of MW1-D2 or 1C2-D2 in combination with the monoclonal ATXN2 antibody (Ataxin2mono-Tb) showed the best discrimination between WT and KI at the concentration of Ataxin2mono-Tb 0.5 ng/μl × MW1-D2 3 ng/μl (labeled in green, supplementary information figure 1a) or with Ataxin2mono-Tb 0.5 ng/μl × 1C2-D2 10 ng/μl (labeled in green, supplementary information figure 1b). In general, TR-FRET signals were lower when using the monoclonal ATXN2 antibody compared to the polyclonal ATXN2 antibody.

### Establishment of Buffer Conditions

To optimize lysis buffer conditions to best detergent (e.g., TritonX, Igepal) and ionic strength (NaCl concentration), three often used lysis buffers (RIPA, PBS and TES/TNES) were compared (Fig. 1d, e) for the antibody combinations ataxin2poly-Tb x MW1-D2 (Fig. 1d) and ataxin2poly-Tb × 1C2-D2 (Fig. 1e), each at the best antibody concentrations as established for RIPA buffer in Fig. 1b, c, for PBS buffer in supplementary information figures 1c+d and for TES/TNES buffer in supplementary information figures 1e+f. Protein homogenates in RIPA buffer reached the highest signals and the best discrimination between WT and KI. Therefore, all further experiments were carried out in RIPA buffer.

### Establishment of Protein Amount

After establishing the antibody combinations, concentrations, and buffer conditions, recombinant ATXN2 isolated from HEK293T cells using Myc-tagged ATXN2 constructs and anti-c-myc agarose were used to prepare a standard curve with a concentration range from 10 to 0.0001 μg/μl recombinant ATXN2 protein with either 22Q or 79Q. TR-FRET immunoassay validation demonstrated a higher specificity for polyQ-expanded ATXN2 (79Q) over normal ATXN2 (22Q, Fig. 1f). Determination of LOD (limit of detection, 7.995 ng/μl) and LOQ (limit of quantification, 26.65 ng/μl) showed a linear detection threshold from 1 ng to 1 μg of recombinant ATXN2 79Q (Fig. 1f). Additionally, a standard curve generated using homogenates from HEK293T cells overexpressing Myc-22Q (red; 22Q) or Myc-79Q (blue; 79Q) revealed also a higher specificity for polyQ-expanded ATXN2 over normal ATXN2 as well as a linear detection range of 10 ng to 10 μg whole protein amount (Fig. 1g). To determine the best total protein concentration for further TR-FRET measurements, MEF homogenates including 2 or 1 μg/μl whole protein concentration isolated from *Atxn2-*CAG100 knock-in mice (blue; KI) or wild-type controls (red; WT) were evaluated (supplementary information figure 2a). Additionally, homogenates (2 or 1 μg/μl) from HEK293T cells transfected with myc ATXN2 plasmids including myc-22Q (red; 22Q) and myc-79Q (blue; 79Q) or empty plasmid (black; Ø) (supplementary information figure 2b) including 2 μg/μl or 1 μg/μl whole protein concentration were used. Samples were lysed in RIPA buffer and measured with the antibody combination ataxin2poly 0.3 ng/μl × MW1-D2 3 ng/μl. Results for the antibody combination Ataxin2mono-Tb 0.5 ng/μl × MW1-D2 3 ng/μl are also shown in the supplement (supplementary information figures 2c+d). Overall, measurements demonstrated that a lower total protein concentration of 1 μg/μl showed higher polyQ-expanded ATXN2-specific signals and a better discrimination between WT and KI samples.

In summary, the polyclonal ATXN2 antibody ataxin2poly-Tb discriminated better between ATXN2 WT and KI samples in comparison to the monoclonal antibody Ataxin2mono-Tb. Therefore, further experiments were performed either in the combination ataxin2poly-Tb x MW1-D2 with the antibody concentration 0.3 ng/μl × 3 ng/μl or in the combination ataxin2poly-Tb x 1C2-D2 with antibody concentration of 0.3 ng/μl × 10 ng/μl. Additionally, RIPA buffer and a total protein concentration of 1 μg/μl were used in all further measurements.

### SCA2 Specificity

As mentioned before, SCA2 belongs to the group of polyQ diseases including SCA1, 3, 6, 7, HD, DRPLA, and SBMA. In each of these diseases, neurodegeneration is mainly triggered by translation of disease proteins with an elongated polyQ region that can potentially be detected by the polyQ-specific antibodies MW1 and 1C2. For the development of an immunoassay to quantify soluble polyQ-expanded protein ATXN2 in human biomaterials, it is therefore essential to demonstrate that no other polyQ proteins are detectable. For that reason, we performed a specificity experiment including cerebellar homogenates from three wildtype controls and SCA2, SCA3, SCA17, and HD mouse models. The antibody combinations ataxin2poly-Tb 0.3 ng/μl × MW1-D2 3 ng/μl and ataxin2poly-Tb 0.3 ng/μl × 1C2-D2 10 ng/μl specifically measured polyQ-expanded disease protein only in the SCA2 samples (supplementary information figures 2e+f). As observed before, ATXN2 values were higher if measured with ataxin2poly-Tb × MW1-D2, but demonstrated also low background signals in controls and in other polyQ diseases.

### Detection of Small Changes in ATXN2 Expression Levels Using siRNA and Starvation Experiments

**Fig. 2:**
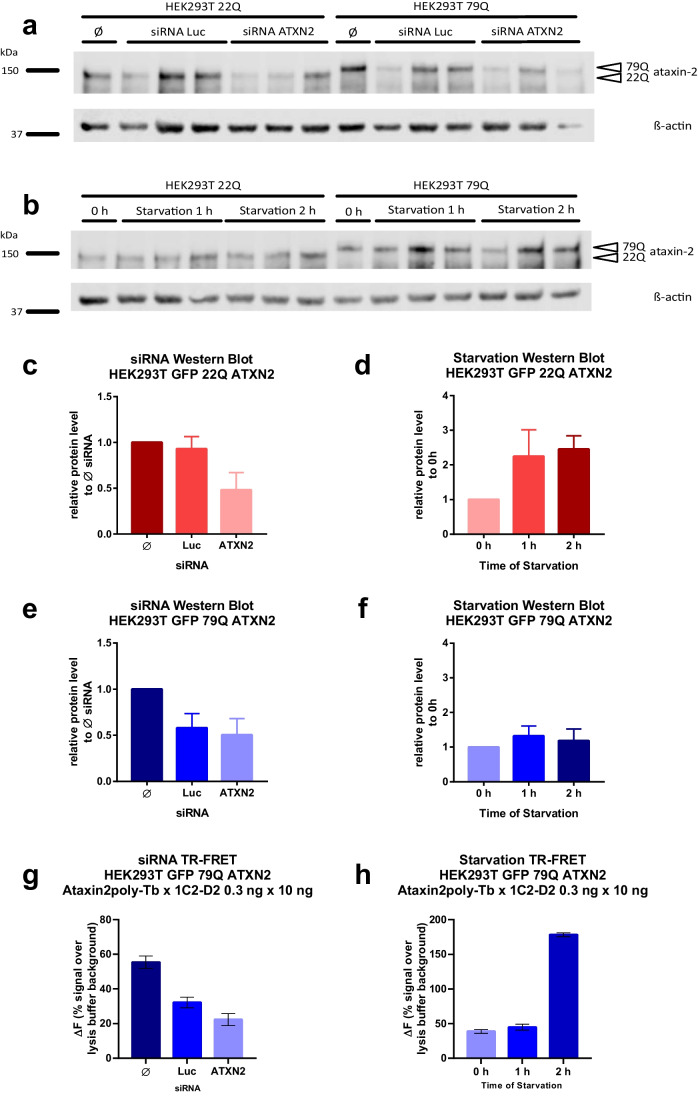
siRNA and starvation experiments demonstrated that the TR-FRET-based immunoassay detects small changes in ATXN2 expression levels. To lower ATXN2 expression, HEK293T cells were transfected with GFP ATXN2 plasmids with 22Q or 79Q and treated either with ATXN2 siRNA (ATXN2), luciferase siRNA (Luc) as a control or without siRNA (Ø) (siRNA experiments). Additionally, GFP ATXN2 transfected HEK293T cells were incubated in HBSS for 0, 1, or 2 h to induce starvation, and therefore, ATXN2 upregulation **a**, **b** Western blot of siRNA experiments (**a**) and western blot of starvation experiments (**b**) using the polyclonal ataxin2poly antibody detected the ATXN2 protein at 150 kDa. β-actin is shown as loading control. (Western Blot analysis with 30 μg of total protein and 8% Bis-Tris gel) **c**–**f** Western blot quantification of ATXN2 expression after lowering by siRNA (**c**, **e**) or ATXN2 increased expression by starvation (**d**, **f**). The bars show the relative protein levels compared to treatment without siRNA (Ø) or 0 h of starvation, respectively. **g**, **h** TR-FRET analysis of the siRNA and starvation experiments using the antibody combination ataxin2poly-Tb 0.3 ng/μl × MW1-D2 10 ng/μl, diluted in RIPA buffer.

As the primary reason to develop an immunoassay is to monitor therapeutic studies, the assay should be able to detect even small protein changes. Therefore, we downregulated ATXN2 expression in HEK293T cells using siRNA and increased polyQ-expanded ATXN2 levels by starvation experiments. For downregulation, HEK293T cells were transfected with GFP ATXN2 plasmids expressing 22Q or 79Q and treated with either ATXN2 siRNA (ATXN2), or as control reference with luciferase siRNA (Luc), or without siRNA (Ø, untreated). For starvation experiments, HEK293T cells transfected with GFP ATXN2 plasmids expressing 22Q or 79Q were grown in HBSS medium for 1 or 2 h to induce starvation. Western blot analyses detected the ATXN2 protein at 150 kDa using the polyclonal ataxin2poly antibody (Fig. 2a, b). Quantification of the ATXN2 22Q or 79Q protein expression under ATXN2 siRNA treatment revealed a slight downregulation compared to untreated or luciferase siRNA-treated cells (Fig. 2c, e). Additionally, inducing starvation revealed increased ATXN2 expression over time (Fig. 2d, f). Measuring the same samples as analyzed by western blot with the TR-FRET immunoassay revealed a clear downregulation of ATXN2 after ATXN2 siRNA treatment (Fig. 2g). The TR-FRET immunoassay analysis of the starvation experiments with 0, 1, or 2 h of starvation in HBSS medium detected an upregulation after 2 h of starvation using the antibody combination ataxin2poly-Tb 0.3 ng/μl × 1C2-D2 10 ng/μl (Fig. 2h).

### ATXN2 Protein Level in Human Material

**Fig. 3:**
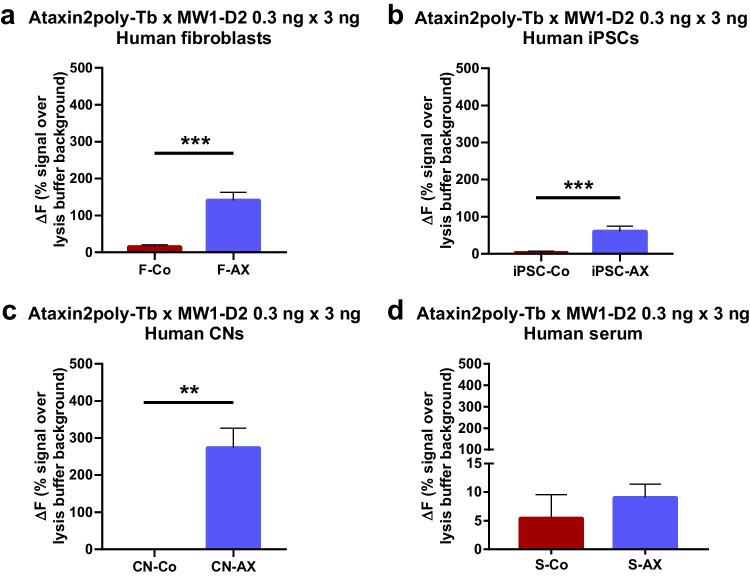
TR-FRET analysis in human cell culture materials. **a** Determination of polyQ-expanded ATXN2 by TR-FRET-based immunoassay in homogenates of human fibroblasts isolated from SCA2 patients *n* = 3 (red; F-AX) and healthy controls *n* = 4 (blue; F-Co). **b** Homogenates of human iPSCs from SCA2 patients *n* = 4 (red; iPSC-AX) compared to *n* = 4 healthy controls (blue; iPSC-Co). **c** Homogenates of human CNs from SCA2 patients *n* = 2 (red; CN-AX) and healthy controls *n* = 4 (blue; CN-Co). **d** Human serum from SCA2 patients *n* = 4 (red; S-AX) compared to *n* = 5 healthy controls (blue; S-Co). All samples (except serum samples) were diluted in RIPA buffer, evaluated with a total protein concentration of 1 μg/μl and analyzed using the antibody combination ataxin2poly-Tb 0.3 ng/μl × MW1-D2 3 ng/μl. **P ≤ 0.01; ***P ≤ 0.001

To assess if the developed immunoassay also measures human soluble polyQ-expanded ATXN2 specifically in human cell lines, different human cell lines including human fibroblasts (Fig. 3a) from SCA2 patients *n* = 3 (red; F-AX) and controls *n* = 4 (blue; F-Co), human iPSCs (Fig. 3b) from SCA2 patients *n* = 4 (red; iPSC-AX) and healthy controls *n* = 4 (blue; iPSC-Co), and human CNs (Fig. 3c) from SCA2 patients *n* = 2 (red; iCN-AX) and healthy controls *n* = 4 (blue; CN-Co) were diluted in RIPA buffer and tested with a total protein concentration of 1 μg/μl with the antibody combination ataxin2poly-Tb 0.3 ng/μl × MW1-D2 3 ng/μl. Our data showed that the newly developed TR-FRET immunoassay is capable to detect specifically soluble polyQ-expanded ATXN2 in patient-derived SCA2 biomaterials compared to respective controls. Western blot analyses confirmed ATXN2 expression in different cell lines (supplementary information figure 2g). Additionally, to determine if the established ATXN2 immunoassay is sensitive enough to measure ATXN2 protein levels in human biofluids, serum samples from 4 SCA2 mutation carrier as well as 5 neurologically healthy controls were analyzed using the antibody combination ataxin2poly-Tb 0.3 ng/μl × MW1-D2 3 ng/μl. Undiluted and 1:2 and 1:4 diluted serum samples were determined, and all measurements revealed lower protein expression values as calculated by the LOQ in both healthy controls and SCA2 patients (Fig. 3d).

## Discussion

In this study, we developed the first sensitive TR-FRET-based immunoassay to measure the soluble polyQ-expanded protein ATXN2 in cellular and animal models as well as human neuronal cell culture.

There is currently no curative therapy for the trinucleotide repeat disease SCA2. One main reason for this is the lack of easily accessible, objective, and sensitive outcome parameters to track disease progression but also the lack of effective molecular target treatments. Unfortunately, clinical read outs such as the Scale for the Assessment and Rating of Ataxia (SARA) need long observation periods due to slow disease progression, show substantial day to day variability and inter-rater differences and rare prone to unspecific changes, e.g., due to injuries. For that reason, molecular markers that respond quickly to therapeutics and are readily detectable in biomaterials such as CSF or peripheral blood are of major importance for monitoring on-target effects for upcoming clinical trials. Recently, it has been shown that the protein tau has increased levels in CSF of SCA2 patients [37], and the protein neurofilament light (NfL) chain is suitable to indicate axonal degeneration in many neurodegenerative diseases, including SCA2 [34, 65]. Therapeutic strategies that reduce specifically polyQ-expanded protein levels are currently being applied to slow or stop disease progression in polyQ diseases like SCA1 [66, 67], SCA2 [68], HD [69, 70], and SCA3 [71–74]. Therefore, the disease proteins itself could serve as an excellent and primary potential therapeutic biomarker to monitor disease protein lowering by, e.g., antisense oligonucleotides. In HD [41–43] and SCA3 [44, 45], immunoassays based on the TR-FRET technology were already established to quantify the respective disease protein in human leukocytes, mononuclear cells (PBMCs), or human tissue samples. Later, the TR-FRET-based immunoassays were further developed into ultra-sensitive single-molecule counting (SMC) immunoassays in order to be able to quantify fmol protein amounts in CSF and peripheral blood of HD and SCA3 mutation carrier, too [46, 47]. Following its successful establishment in HD and SCA3, the soluble polyQ-expanded ATXN2 protein should also be validated as potential prognostic and therapeutic biomarker in SCA2.

To establish a new TR-FRET-based immunoassay for ATXN2, two antibodies which bind specifically to the disease protein are required. As shown for HD and SCA3, specific detection of the polyQ-expanded disease protein can be achieved by using one polyQ-specific antibody, like MW1. In our study, two different polyQ-specific antibodies were tested as acceptor antibodies: MW1 and 1C2. Importantly, both polyQ-specific antibodies are shown to have similar binding characteristics, which display similar binding properties with polyQ of various lengths [75]. To achieve SCA2 specificity, two ATXN2-specific antibodies were tested as donor antibody including a polyclonal ATXN2 antibody and a monoclonal ATXN2 antibody. Determination of best antibody pair and conditions revealed better results for the polyclonal ATXN2 antibody in combination with the polyQ-specific antibodies MW1 and 1C2. Following further optimizations, all biomaterials were subsequently lysed in RIPA buffer, biomaterial prediluted to a total protein concentration of 1 μg/μl, and measurements performed after an incubation of biomaterial to antibody mixture for 24 h at 4 °C.

Since the polyQ-specific antibodies MW1 or 1C2 can also detect other proteins with an expanded polyQ region, we proved that the newly established ATXN2 immunoassay quantifies specifically the soluble polyQ-expanded ATXN2 protein. As shown already similarly for HD and SCA3, our newly established ATXN2 immunoassay specifically quantifies soluble polyQ-expanded ATXN2 if the polyQ-specific antibody MW1 is combined with an ATXN2-specific donor antibody [44].

In order to use the protein ATXN2 as a molecular readout parameter for disease progression and potentially in clinical trials which aim to reduce specifically the disease protein (“protein lowering therapies”), the immunoassay must be able to detect small protein changes. Therefore, down- and upregulated ATXN2 using siRNA and starvation experiments were performed and demonstrated that the polyQ-expanded ATXN2 immunoassay can reliably detect small protein changes.

To demonstrate target engagement in future trials that aim at silencing SCA disease genes, the availability of sensitive immunoassays to measure the concentration of polyQ-expanded disease proteins like ATXN2 in body fluids is mandatory [76]. Therefore, we confirmed that the new TR-FRET-based immunoassay is able to measure polyQ-expanded ATXN2 in human biomaterials like patient-derived cell lines including human fibroblasts, induced pluripotent stem cells, and iPSC-derived cortical neurons. Importantly, in all patient-derived cell lines, the immunoassay perfectly discriminated between SCA2 patients and healthy controls. However, the aim is to adapt the immunoassay to human biomaterials like cerebrospinal fluid (CSF) or peripheral blood. Unfortunately, first measurements in human SCA2 patient serum samples revealed that the level of expanded ATXN2 is lower as the determined immunoassay LOQ and therefore not high enough for evaluation with the newly developed TR-FRET-based immunoassay. As known for HD and SCA3, significantly lower concentrations of respective disease proteins are presumably present in the CSF or in peripheral blood [46, 47]. ATXN2 is a ubiquitous expressed protein, with high mRNA expression in all brain areas (e.g., cerebellum 33.10 TPM, transcript per million), but also hearth (11 TPM), kidney (10.28 TPM), lung (24.26 TPM), liver (8.950 TPM), and cultured fibroblasts with 15.98 TPM (https://gtexportal.org/home/gene/ENSG00000204842.14, [77]). The lowest expression is demonstrated for whole blood with 4.048 TPM, which explains why the polyQ-expanded ATXN2 immunoassay can measure successfully in patient-derived cell lines (cultured fibroblasts, 15.86 TPM) but not in serum isolated from whole blood (4.048 TPM) (https://gtexportal.org/home/gene/ENSG00000204842.14, [77]). ATXN2 is shown to increase to nutrient and trophic stress in a mTOR-signaling–dependent manner [78]. Therefore, it could be an option to perform a “fasting blood test” (no eat and drink for at least 8 h before blood take) to increase ATXN2 expression in whole blood and therefore may be able to increase ATXN2 expression to a measurable level by the newly developed TR-FRET-based assay.

Additionally, with the successfully established polyQ-expanded ATXN2 TR-FRET-based immunoassay, we have now the tool available to adapt the assay to an ultrasensitive platform like SMC or SIMOA in the future. As shown for HD and SCA3 that is important, because of very low–soluble protein levels in CSF, peripheral blood including serum and plasma [46, 47]. On mRNA level, HTT (huntingtin, 5.990 TPM), ATXN2 (4.048 TPM), and ATXN3 (0.8812 TPM) show only very low expression in whole blood compared to α-synuclein (SNCA) which demonstrate a high expression of 33.48 TPM (https://gtexportal.org/home/gene/ENSG00000204842.14, [77]). In line with that, no TR-FRET for measurements in whole blood or CSF could be established for HTT, ATXN3, or ATXN2 but showed positive results for SNCA [41–45, 79].

As indicated, the new ATXN2 immunoassay is with an assay range of around 1 ng to 10 μg more sensitive as other known techniques to determine protein expression including western blot analyses. But as most techniques, also this kind of immunoassay show some limitations including ran-translation [80], post-translational protein modifications [81], but also ATXN2 alternative splicing variants [82] or genomic mutations [48, 57, 83], which may interfere with the antibody binding sites. ATXN2 is shown to modulate different neurodegenerative diseases like ALS or SCA3 by intermediate repeat length [48]. Both, MW1 and 1C2 polyQ-specific antibodies, are characterized to bind glutamines of different length starting with 11Q but demonstrated higher affinity to longer repeats, because the number of epitopes is increasing with polyQ length (e.g., in the case of MW1, it increases from 13 epitopes in 22Q to 32 epitopes in 41Q) [75]. For SCA3 and HD, established immunoassays using the MW1 antibody results in the specific measurement of soluble polyQ-expanded disease protein and not determine controls with intermediated repeat length [46, 47]. To test that fact also for the newly developed soluble polyQ-expanded immunoassay ATXN2, we analyzed 5 PBMC samples from individuals with intermediated repeat length of 27 to 30 glutamines compared to individuals with 22Q. The measurements revealed lower signals as the determined immunoassay LOQ in both genotypes (data not shown) and might be explained by the fact that the multiplicity of epitopes of the polyQ-specific antibodies, combined with the possibility of several antigen-binding domain interacting simultaneously on longer polyQ, constitute an unusually complex interaction. Therefore, conditions are present to detect only the longer polyQ, even when similar quantities of short and long polyQ are present [75].

In summary, we described the first ATXN2 immunoassay to measure specifically soluble polyQ-expanded ATXN2 in cellular and animal tissue as well as in human cell lines. Further validation in human biomaterials like CSF, peripheral blood, or PBMCs, including blood take under fasting conditions and correlation to clinical data, is needed to confirm polyQ-expanded ataxin-2 as new important molecular readout parameter in further patient-based studies.

## Supplementary Information

ESM 1:

## Data Availability

All data generated during this study are included in this article or are available on reasonable request from the corresponding author.

## Abbreviations

1C2-D2: PolyQ-specific antibody 1C2, D2 labeled
AD: Alzheimer’s disease
ALS: Amyotrophic lateral sclerosis
Ataxin2mono-Tb: Monoclonal ATXN2 antibody, Tb labeled
Ataxin2poly-Tb: Polyclonal ATXN2 antibody, Tb labeled
ATXN2: Ataxin-2 (protein)
Ax: SCA2 patient
CN: iPSC-derived cortical neuron
Co: Control patient
CSF: Cerebrospinal fluid
DRPLA: Dentatorubral-pallidoluysian atrophy
ex: Expanded
FBS: Fetal bovine serum
FTD: Frontotemporal dementia
HBSS: Hanks’ Balanced Salt Solution
HD: Huntington’s disease
HEK: Human embryonic kidney
iPSC: Induced pluripotent stem cell
KI: Knock-in
LOD: Limit of detection
LOQ: Limit of quantification
Luc: Luciferase
MEF: Mouse embryonic fibroblast
MW1-D2: PolyQ-specific antibody MW1, D2 labeled
NfL: Neurofilament light
PBMC: Peripheral blood mononuclear cells
polyQ: Polyglutamine
Q: Glutamine
RT: Room temperature
SARA: Scale for the Assessment and Rating of Ataxia
SBMA: Spinal and bulbar muscular atrophy
SCA: Spinocerebellar Ataxia
SCA2: Spinocerebellar Ataxia Type 2
SEM: Standard error of the mean
SMC: Single-molecule counting
SNCA: α-synuclein
TPM: Transcript per million
TR-FRET: Time-resolved fluorescence energy transfer
TTR-FAP: Transthyretin familial amyloid polyneuropathy
WT: Wildtype
