# Supplementary material for: TR-FRET-Based Immunoassay to Measure Ataxin-2 as a Target Engagement Marker in Spinocerebellar Ataxia Type 2
Source: Mol Neurobiol. 2023 Mar 9;60(6):3553–67. doi: 10.1007/s12035-023-03294-y (PMC10122633; doi:10.1007/s12035-023-03294-y)
Supplement: Supplementary file 1 — ESM 1: [file 12035_2023_3294_MOESM1_ESM.docx]

Supplementary information Fig. 1: Establishment of antibody combination and determination of the optimal antibody concentrations: **a+b**) Combination of the antibodies Ataxin2mono-Tb and MW1-D2 (**a**) or Ataxin2mono-Tb and 1C2-D2 (**b**) each in three different concentrations: Ataxin2poly-Tb with 0.3 ng, 0.5 ng and 1 ng/µl, MW1- D2 or 1C2-D2 with 1 ng, 3 ng and 10 ng/µl. Conduction of the TR-FRET measurement with homogenates from wildtype mouse liver (red; WT) and Atxn2-CAG100 knock-in mouse liver (blue; KI), a total protein concentration of 1 µg/µl in RIPA buffer. The highest discrimination between WT and KI (green box) was achieved with the concentrations of Ataxin2mono-Tb 0.5 ng/µl x MW1-D2 3 ng/µl (**a**) and Ataxin2mono-Tb 0.3 ng/µl x 1C2-D2 10 ng/µl (**b**). Comparison of different lysis buffers: **c-f**) Combination of the antibodies Ataxin2poly-Tb and MW1-D2 (**c+e**) or Ataxin2poly-Tb and 1C2-D2 (**e+f**) each in three different concentrations: Ataxin2poly-Tb with 0.3 ng, 0.5 ng and 1 ng/µl, MW1- D2 or 1C2-D2 with 1 ng, 3 ng and 10 ng/µl. Conduction of the TR-FRET measurement with homogenates from wildtype mouse liver (red; WT) and Atxn2-CAG100 knock-in mouse liver (blue; KI), a total protein concentration of 1 µg/µl in PBS buffer (**c+d**) or in TES/TNES buffer (**e+f**). The highest discrimination between WT and KI is illustrated by a green box.

Supplementary information Fig. 2: **a-d)** Testing of different protein concentrations. Homogenates from mouse embryonic fibroblasts (MEF) isolated from wildtype mice (red; WT) or Atxn2-CAG100 knock-in mice (blue; KI) (**a+c**) or homogenates from HEK293T (HEK) cells, which were either transfected with myc ATXN2 plasmids with a length of 22 glutamines (red; 22Q), a length of 79 glutamines (blue; 79Q) or empty myc plasmids (black; Ø) (**b+d**) were diluted in RIPA buffer to a total protein concentration of 2 µg/µl or 1 µg/µl and measured with the antibody combination Ataxin2poly-Tb 0.3 ng/µl x MW1-D2 3 ng/µl **(a+b)** or Ataxin2mono-Tb 0.5 ng/µl x MW1-D2 3 ng/µl **(c+d)** using TR-FRET. **e+f)** The SCA2 specificity was determined with the antibody combinations Ataxin2poly-Tb x MW1-D2 **(e)** and Ataxin2poly-Tb x 1C2-D2 **(f)**. For this purpose, homogenates from wildtype mouse cerebellum (red; control), SCA3, SCA17 or HD mouse cerebellum homogenates (all green) compared to SCA2 Atxn2-CAG100 knock-in mouse cerebellar samples (blue; SCA2) were measured with the indicated antibody combination and concentrations. Both antibody combinations can specifically distinguish the SCA2 samples. **g)** Western Blot of human material: lysates of human fibroblasts, human induced pluripotent stem cells (iPSCs) and human cortical neurons (CNs) from SCA2 patients (Ax) and matching controls (Co) detected with the polyclonal Ataxin2poly antibody. β-Actin is shown as loading control. The protein ATXN2 can be detected at 150 kDa (Western Blot analysis with 20 µg of total protein and 10% Bis-Tris gel; ex = expanded ataxin-2, WT = wildtype ataxin-2).

Supplementary information Fig. 3: Full western blot images.

**a+b)** Western blots of siRNA experiments (Fig. 2a). To lower ATXN2 expression, HEK293T cells were transfected with GFP ATXN2 plasmids with 22Q or 79Q and treated either with ATXN2 siRNA (ATXN2), luciferase siRNA (Luc) as a control or without siRNA (Ø). Using the polyclonal Ataxin2poly antibody, ATXN2 protein was detected at around 150 kDa with a size shift between 22Q and 79Q ATXN2. **(a)**. β-actin is shown as loading control **(b)**. An 8% Bis-Tris gel was loaded with 30 µg total protein.

**c+d)** Western blots of starvation experiments (Fig. 2b). GFP ATXN2 (22Q, 79Q) transfected HEK293T cells were incubated in HBSS for 0, 1 or 2 hours to induce starvation and therefore, ATXN2 upregulation. The polyclonal Ataxin2poly antibody detected the ATXN2 protein at around 150 kDa **(c)**. β-actin is shown as loading control **(d)**. An 8% Bis-Tris gel was loaded with 30 µg total protein.

**e+f)** Western blots of human material (supplementary information Fig. 2e): lysates of human fibroblasts, human induced pluripotent stem cells (iPSCs) and human cortical neurons (CNs) from SCA2 patients (Ax) and matching controls (Co) detected with the polyclonal Ataxin2poly antibody **(e)**. β-Actin is shown as loading control **(f)**. An 10% Bis-Tris gel was loaded with 20 µg total protein.; ex = expanded ataxin-2, WT = wildtype ataxin-2).
